# A Prospective Randomised Controlled Study Comparing Ultrasonic Dissector with Electrocautery for Axillary Dissection in Patients of Carcinoma Breast

**DOI:** 10.21315/mjms2021.28.1.12

**Published:** 2021-02-24

**Authors:** Ananya Deori, Nikhil Gupta, Arun Kumar Gupta, Raghav Yelamanchi, Himanshu Agrawal, C K Durga

**Affiliations:** Department of Surgery, Atal Bihari Vajpayee Institute of Medical Sciences and Dr Ram Manohar Lohia Hospital, New Delhi, India

**Keywords:** modified radical mastectomy, axillary dissection, ultrasonic dissector, monopolar electrocautery, post-mastectomy seroma

## Abstract

**Background:**

Axillary dissection is one of the important components of modified radical mastectomy (MRM). The present study was conducted to compare surgical outcomes by using monopolar electrocautery and ultrasonic dissector for axillary dissection in MRM.

**Methods:**

A parallel randomised controlled single blinded study was conducted with a sample size of 70 patients who were randomised into two groups. One group underwent MRM using ultrasonic dissector (Group A) and the other one using electrocautery (Group B). Intra- and post-operative outcomes were compared.

**Results:**

Group A had an average operating time of 30.86 min, which was statistically less than that of Group B. The mean mop count and the daily drain output in Group A were less as compared to Group B and the differences were statistically significant. Drain was removed early in Group A as compared to Group B. However, post-operative pain scores and seroma formation were not statistically significant among the two groups.

**Conclusion:**

Ultrasonic dissector group had significantly lesser intra-operative bleeding, operating time and post-operative drain output when compared to electrocautery group. However, the two groups had no significant difference in post-operative pain scores and seroma formation.

## Introduction

Breast cancer is the most common malignancy among women in India and across the globe (1). The incidence of breast cancer is steadily rising, especially in the developing countries (2). Surgery remains the main stay of treatment for non-metastatic breast cancer and has evolved from Halsted radical mastectomy to modified radical mastectomy (MRM) and further to breast conservative surgery (3). Axillary dissection is one of the important components of breast cancer surgery and can be performed by a variety of techniques using scalpel, scissors, electrocautery or ultrasonic dissector.

Seroma formation is a common complication following MRM and a variety of hypotheses have been proposed for its etiology. These include the presence of dead space post-resection, surgical disruption of lymphatic pathways, thermal damage to the lymphatics and inflammatory exudates (4–6). Seroma formation also depends on the age and body mass index (BMI) of patients (7). The amount of serous discharge ultimately determines the duration of post-operative drains, hospital stay and cost of medical care. Hence, this has been an area of intense research with the aim to reduce post-operative serous discharge by variety of techniques.

Cold knife was commonly used before the advent of energy devices. Dissection with cold knife results in more blood loss when compared to energy devices. However, it does not cause any damage to the surrounding tissues. Monopolar electrocautery is being widely used for dissections and homeostasis over several decades. Studies have shown that dissection using monopolar electrocautery results in a higher incidence of seroma formation due to thermal damage to the lymphatics (8). However, it is advantageous in reducing intraoperative blood loss and operating time (9).

Ultrasonic dissector has blades that vibrate at a frequency of 55,500 Hz, thereby converting electrical energy into mechanical energy and resulting in protein coagulation due to breakage of hydrogen bonds. This leads to cutting and coagulation of tissues and subsequent sealing of vascular and lymphatic capillaries. It causes less heat dispersion when compared to monopolar electrocautery, thereby resulting in less tissue damage (10). It has an added advantage of reduced smoke emission and absence of charring of tissues (11). However, it is more costly than monopolar electrocautery.

The present study was conducted to compare the surgical outcomes by using monopolar electrocautery and ultrasonic dissector for axillary dissection in MRM. Authors have hypothesized that the use of ultrasonic dissector for axillary dissection in MRM results in better surgical outcomes as it causes less thermal damage to surrounding tissues and smoke production with good vessel sealing property.

## Methods

Institutional ethics committee approval for the study was taken prior to the study commencement as it involved human participants. All patients were enrolled in the study after taking written informed consent.

### Study Design and Population

A parallel randomised controlled single blinded study was conducted in our tertiary care and academic center located in India from November 2014 to March 2016. Sample size of the study was 70 patients who were divided equally into two groups. All female patients, who presented to the surgical department of our hospital and were planned for modified radical mastectomy for operable breast carcinoma, were invited to participate in the study irrespective of their neoadjuvant chemotherapy status. Patients were randomised into two groups by opaque sealed envelope method before axillary dissection either to undergo axillary dissection using ultrasonic dissector or using monopolar electrocautery (Group B). Patients were blinded to the method used for axillary dissection. In the post-operative period, drain output was monitored. Post-operative pain was measured and monitored on visual analogue scale (VAS). Patients were followed up for a period of 30 days for any complications in the post-operative period.

### Inclusion Criteria

All female patients diagnosed with carcinoma breast who were planned for modified radical mastectomy and consented to participate in the study were enrolled in the study irrespective of their previous neoadjuvant chemotherapy status.

### Exclusion Criteria

Patients with metastatic breast carcinoma, patients undergoing breast conservation surgery or immediate breast reconstruction procedure, pregnant females and those with prior history of axillary surgeries were excluded from the study. Also, patients with a history of any coagulopathy or those using any antiplatelet or anticoagulant medications were also excluded from the study.

### Management

Patients underwent Auchincloss-modified radical mastectomy under general anaesthesia. The same surgical team consisting of one senior surgeon, one senior resident and one junior resident operated all the cases to ensure uniformity of the technique. Skin incision was made with a scalpel and, superior and inferior flaps were dissected using electrocautery. The breast tissue along with the underlying pectoralis fascia was dissected from the medial to lateral side. Just before dissecting the clavipectoral fascia and entering the axilla, an opaque sealed envelope was opened and the method of dissection of axilla was decided. Ultrasonic dissector (Hormonic Focus, Ethicon Inc, USA) was used for axillary dissection in Group A. Monopolar electrocautery (Megadyne, Ethicon Inc, USA) was used for dissection in Group B.

Scrub nurse was asked to make a separate count of mops used for axillary dissection. Once a mop was fully soaked, it was discarded. All fat, fascia and level I and II axillary lymph nodes were removed in all cases with preservation of the long thoracic nerve and thoracodorsal pedicle. Thereafter, homeostasis was achieved. Two drains were placed, one in the axilla and the other beneath the flaps and were connected to two separate suction drains. Flaps were approximated using suture and compression dressing was applied.

Patients received routine post-operative care. Drain output was monitored daily and were emptied once every 24 h and suction was reapplied. Drains were removed when the output was < 30 mL per day on two consecutive days. Post-operative pain was monitored using VAS on a scale from 0 to 10 twice daily till postoperative day (POD) 5. Patients were discharged when both the drains were removed and when no undue complications were present. All patients were followed up in the outpatient department for a period of 30 days. The outcomes studied were:

- Operative time for axillary dissection (in minutes)
- Intra-operative blood loss during axillary dissection
- Post-operative pain score using VAS iv) Axillary drain volume (in mL)
- Duration of axillary drain (in days)
- Post-operative complications like seroma formation, wound infection, hematoma and flap necrosis

## Statistical Analysis

Total sample size was 70 patients calculated with a margin error of 5% and were distributed equally into two groups. The data acquired was coded and recorded in an MS Excel spreadsheet (Microsoft Office, Microsoft, Washington). Data was analysed using Statistical Package for Social Sciences (SPSS) version 21.0 (IBM SPSS Statistics, International Business Machines Corporation, New York) and was normally distributed. The comparison of normally distributed continuous variables between the groups was performed using Student’s *t*-test. Nominal categorical data between the groups were compared using Chi-square test or Fisher’s exact test as appropriate. *P*-value of < 0.05 was considered statistically significant.

## Results

The mean age of patients in Groups A and B was 50.5 years old and 51 years old, respectively, which was not statistically different (Table 1). The mean BMI of patients in Groups A and B was 23.6 kg/m^2^ and 23.1 kg/m^2^, respectively, which were statistically not significant (Table 1). The comorbidities of patient population of both the groups are listed in Table 1. The distribution of cases as per the tumour-node-metastasis (TNM) staging in the two groups is shown in Table 2.

Comparing the variables, the time for axillary dissection was much shorter in Group A than Group B. The former had an average operating time of 30.86 min (20 min–44 min), while for Group B, it was 40.63 min (27 min–50 min) (Table 3). This difference was statistically significant (*P* < 0.001). The mean mop count in Groups A and B was 5.51 ± 1.84 and 7.20 ± 1.32, respectively (Table 3). This difference was found to be statistically significant (*P* < 0.001). No intra-operative complications were noted in both the groups.

Post-operative pain between the two groups was not found to be statistically significant. The post-operative drain output between the two groups was also compared. The daily drain output in Group A was low as compared to Group B, and the difference was statistically significant for the first 3 days (Table 4). The cumulative drain output for the first 3 days was also low for Group A in comparison to Group B and the result was statistically significant (Table 3). Axillary drain was removed early in Group A as compared to other group and the result was statistically significant (Table 3).

In Group A, one patient had post-operative flap necrosis, while in Group B, two patients had flap necrosis (Table 5). In both the groups flap necrosis occurred within the first seven days. In Group A, seroma occurred between 8 days and 15 days in one patient. In Group B, one patient had seroma formation within the first 7 days, while two patients had seroma formation between 8 days and 15 days (Table 5). The difference in seroma incidence between Group A and Group B was not statistically significant with a *P*-value of 0.303.

## Discussion

Cold knife was traditionally used for flap and axillary dissection in MRM. Tissue trauma was less with cold knife, and also the flaps had more tensile strength and collagen content (12). However, bleeding was an important complication of cold knife, which negatively impacts intra-operative surgical field and also prolongs surgery duration. Monopolar electrocautery has been introduced decades ago to address haemostasis in surgery. It has drastically reduced intra-operative bleeding and operating time but at the expense of increased surrounding tissue damage due to heat dissipation (13). Studies comparing the inflammatory markers profile obtained from the drain fluid of MRM patients revealed that MRM performed using electrocautery has the highest inflammatory mediators when compared to cold knife and ultrasonic dissector (12). Electrocautery was also associated with higher incidence of seroma formation, flap necrosis and wound infection in these patients (14, 15). Ultrasonic dissector is based on a novel technology of coagulating proteins using vibration rather than with heat. It has been hypothesised that tissue damage with ultrasonic dissector is minimal as its effect is limited to 0.1mm thickness of surrounding tissues (12). The present study was primarily aimed to study the use of ultrasonic dissector for axillary dissection in MRM in comparison to monopolar electrocautery.

Patient population in both the groups was not statistically different with respect to age, BMI and comorbidities in our study. The mean operating time of axillary dissection performed using ultrasonic dissector was significantly less as compared to electrocautery group. This was also proved in a previous study by Archana et al. (14). This can be explained because of the advantage of the smokeless field while using ultrasonic dissector. However, in many studies there was no difference in the operating time while using ultrasonic dissector or monopolar cautery (17, 18). The amount of blood loss during axillary dissection performed using ultrasonic dissector was significantly less when compared to electrocautery in our study. This finding was well proven in previous studies by Archana et al., Huang et al. and Adwani et al. (14, 17, 19). In a meta-analysis of seven studies by Huang et al. (17), the mean blood loss in ultrasonic dissector group was found to be 300 mL, whereas the mean blood loss in electrocautery group was found to be 399 mL. However, in a previous meta-analysis by Currie et al. (18), the mean blood loss in ultrasonic dissector and electrocautery group was 236 mL and 365 mL, respectively. However, it was not statistically significant.

Many factors have been postulated for seroma formation post-MRM (20). Among the multitude of factors proposed, dissection technique has been widely studied. In the present study, the daily axillary drain output was significantly less in ultrasonic dissector group when compared to electrocautery group. This was in line with other previous studies (14, 17–19, 21–23). It has been hypothesised that electrocautery causes substandard lymphatic vessel sealing and also thrombosis of sub-dermal vessels, resulting in more serious fluid output. On the other hand, ultrasonic dissector has superior sealing effect of lymphatic capillaries and also incites lesser immunological reaction due to minimal tissue damage, thereby decreasing the drain output (17). The total duration of drain requirement was also significantly less in ultrasonic dissector group when compared to electrocautery group in the present study similar to many other previous studies.

The difference in the mean VAS scores was not significant among ultrasonic dissector and electrocautery group in our study. In a study by Archana et al. (14), the difference in the VAS score was significant only on POD 1 and not significant on POD 2–5. Although the amount of drain output and duration of drain required was less in ultrasonic dissector group, the difference in the incidence of post-operative seroma formation between the two groups was not significant in our study.

Ultrasonic dissector usage is not without limitations. Cost is an important factor that limits the acceptance of this technique. Electrocautery is five times cheaper than ultrasonic dissector and may hinder the widespread usage of ultrasonic dissector in developing countries. However, the beneficial effects of ultrasonic dissector proven in various studies might be encouraging in changing our practice of performing MRM in spite of higher costs.

The strength of this study was in the study design and randomisation of patients. As a single surgical team operated on all the patients there was no scope for bias due to surgical technique in this study. The main limitation of the study was its small sample size, which limits the wider application of the results of this study. As the study was performed in a single tertiary care centre, there may be centripetal bias. Studies on larger patient groups are required to validate the results of this study on larger populations.

## Conclusion

On comparing ultrasonic dissector with monopolar electrocautery for axillary dissection in MRM, ultrasonic dissector group had significantly lesser intra-operative bleeding and operating time as compared to electrocautery group. Post-operatively, ultrasonic dissector group had significantly lesser drain output and shorter duration of drain requirement as compared to electrocautery group. However, the two groups had no significant difference in the post-operative pain score and seroma formation.

## Figures and Tables

**Table 1:** Age, BMI and comorbidities of study subjects

	Group A	Group B	*P*-value
Age in years	Mean (SD): 50.54 (15.6)	Mean (SD): 51 (15.9)	0.903*
	Range: 28–68	Range: 30–71	
Body mass index (kg/m^2^)	Mean (SD): 23.6 (5.2)	Mean (SD): 23.1 (5.86)	0.709*
	Range: 18.8–32.4	Range: 19.2–29.8	
Hypertension	5	4	> 0.950**
Diabetes	3	2	> 0.950**
Hypothyrodism	2	2	> 0.950**
Diabetes + hypertension	1	1	> 0.950**
Sample size	35	35	

Notes: SD = standard deviation;

* Student *t*-test;

** Fisher’s exact test

**Table 2:** Distribution of cases as per the TNM staging in Groups A and B

Stage	Group A	Group B
Stage IIA	3	2
Stage IIB	12	14
Stage IIIA	7	7
Stage IIIB	12	10
Stage IIIC	1	2

Total	35	35

**Table 3:** Outcomes between the two groups

		Mean (SD)	Mean difference	95% CI	*P*-value
Operating time for axillary dissection (minutes)	Group A	30.86 (5.79)	9.77	6.94–12.59	< 0.001*
	Group B	40.63 (6.07)			
Blood loss (mop count)	Group A	5.51 (1.84)	1.69	0.92–2.45	< 0.001*
	Group B	7.20 (1.32)			
Post-operative pain (VAS)	Group A	5.63 (1.00)	0.17	−0.29–0.63	0.462*
	Group B	5.80 (0.93)			
Total drain output (POD 1–3 in mL)	Group A	161.00 (40.38)	58.00	33.47–82.30	< 0.001*
	Group B	219.00 (60.46)			
Duration of drain (days)	Group A	4.17 (0.45)	0.72	0.38–1.05	< 0.001*
	Group B	4.89 (0.87)			

Notes: SD = standard deviation; CI = confidence interval;

* Student *t*-test

**Table 4:** Daily axillary drain output from POD 1–3

		Mean (SD)	Mean difference	95% CI	*P*-value
Drain output POD 1 (mL)	Group A	101.14 (17.28)	19.43	10.00 to 28.85	< 0.001*
	Group B	120.57 (21.96)			
Drain output POD 2 (mL)	Group A	44.57 (17.38)	22.00	12.24 to 31.70	< 0.001*
	Group B	66.57 (23.13)			
Drain output POD 3 (mL)	Group A	15.74 (8.63)	16.12	9.29 to 22.94	< 0.001*
	Group B	31.86 (18.31)			
Total drain output (POD 1–3 in mL)	Group A	161.00 (40.38)	58.00	33.47 to 82.52	< 0.001*
	Group B	219.00 (60.46)			

Notes:

* Student *t*-test

**Table 5:** Post-operative complications

Complication	Group A		Group B	
	
	Frequency	Proportion	Frequency	Proportion
Flap necrosis				
1–7 days	1	2.9%	2	5.8%
8–15 days	0	0	0	0
16–30 days	0	0	0	0
Seroma formation				
1–7 days	0	0	1	2.9%
8–15 days	1	2.9%	2	5.8%
16–30 days	0	0	0	0

## References

[1] Asthana S, Chauhan S, Labani S (2014). Breast and cervical cancer risk in India: an update. Indian J Public Health.

[2] Rangarajan B, Shet T, Wadasadawala T, Nair NS, Sairam RM, Hingmire SS (2016). Breast cancer: An overview of published Indian data. South Asian J Cancer.

[3] Freeman MD, Gopman JM, Salzberg CA (2018). The evolution of mastectomy surgical technique: from mutilation to medicine. Gland Surg.

[4] Woodworth PA, McBoyle MF, Helmer SD, Beamer RL (2000). Seroma formation after breast cancer surgery: incidence and predicting factors. Am Surg.

[5] Di Monta G, Caracò C, Crispo A, Marone U, Mozzillo N (2012). Collagen sealant patch to reduce lymphatic drainage after lymph node dissection. World J Surg Oncol.

[6] Srivastava V, Basu S, Shukla VK (2012). Seroma formation after breast cancer surgery: what we have learned in the last two decades. J Breast Cancer.

[7] Shanmugam S, Govindasamy G, Hussain SA, Rao PSH (2017). Axillary dissection for breast cancer using electrocautery versus ultrasonic dissectors: A prospective randomized study. Indian J Cancer.

[8] Porter KA, O’Connor S, Rimm E, Lopez M (1998). Electrocautery as a factor in seroma formation following mastectomy. Am J Surg.

[9] Lee D, Jung BK, Roh TS, Kim YS (2020). Ultrasonic dissection versus electrocautery for immediate prosthetic breast reconstruction. Arch Plast Surg.

[10] Faisal M, Fathy H, Shaban H, Abuelela ST, Marie A, Khaled I (2018). A novel technique of harmonic tissue dissection reduces seroma formation after modified radical mastectomy compared to conventional electrocautery: a single-blind randomized controlled trial. Patient Saf Surg.

[11] Weld KJ, Dryer S, Ames CD, Cho K, Hoigan C, Lee M (2007). Analysis of surgical smoke produced by various energy-based instruments and effect on laparoscopic visibility. J Endourol.

[12] Yilmaz KB, Dogan L, Nalbant H, Akinci M, Karaman N, Ozaslan C (2011). Comparing scalpel, electrocautery and ultrasonic dissector effects: the impact on wound complications and pro-inflammatory cytokine levels in wound fluid from mastectomy patients. J Breast Cancer.

[13] Lumachi F, Brandes AA, Burelli P, Basso SM, Iacobone M, Ermani M (2004). Seroma prevention following axillary dissection in patients with breast cancer by using ultrasound scissors: a prospective clinical study. Eur J Surg Oncol.

[14] Archana A, Sureshkumar S, Vijayakumar C, Palanivel C (2018). Comparing the harmonic scalpel with electrocautery in reducing post-operative flap necrosis and seroma formation after modified radical mastectomy in carcinoma breast patients: a double-blind prospective randomized control trail. Cureus.

[15] Agrawal A, Ayantunde AA, Cheung KL (2006). Concepts of seroma formation and prevention in breast cancer surgery. ANZ J Surg.

[16] Galatius H, Okholm M, Hoffmann J (2003). Mastectomy using ultrasonic dissection: effect on seroma formation. Breast.

[17] Huang J, Yu Y, Wei C, Qin Q, Mo Q, Yang W (2015). Harmonic scalpel versus electrocautery dissection in modified radical mastectomy for breast cancer: a meta-analysis. PLoS One.

[18] Currie A, Chong K, Davies GL, Cummins RS (2012). Ultrasonic dissection versus electrocautery in mastectomy for breast cancer — a meta-analysis. Eur J Surg Oncol.

[19] Adwani A, Ebbs SR (2006). Ultracision reduces acute blood loss but not seroma formation after mastectomy and axillary dissection: a pilot study. Int J Clin Pract.

[20] Chintamani, Singhal V, Singh J, Bansal A, Saxena S (2005). Half versus full vacuum suction drainage after modified radical mastectomy for breast cancer- a prospective randomized clinical trial[ISRCTN24484328]. BMC Cancer.

[21] Sanguinetti A, Docimo G, Ragusa M, Calzolari F, D’Ajello F, Ruggiero R (2010). Ultrasound scissors versus electrocautery in axillary dissection: our experience. G Chir.

[22] Khan S, Khan S, Chawla T, Murtaza G (2014). Harmonic scalpel versus electrocautery dissection in modified radical mastectomy: a randomized controlled trial. Ann Surg Oncol.

[23] Anlar B, Karaman N, Dogan L, Ozaslan C, Atalay C, Altinok M (2013). The effect of harmonic scalpel, electrocautery, and scalpel use on early wound complications after modified radical mastectomy. European Surgery.

